# Integrative analysis reveals prognostic value of cuproptosis and copper hemostasis related genes in immunotherapy for non-small cell lung cancer

**DOI:** 10.1038/s41698-025-01138-7

**Published:** 2025-11-20

**Authors:** Dong Dong, Yaxin Wang, Tong Lu, Yichao Han, Liqiang Shi, Yuqin Cao, Jiahao Zhang, Yajie Zhang, Hecheng Li

**Affiliations:** Department of Thoracic Surgery, Ruijin Hospital, Shanghai Jiao Tong University School of Medicine, Shanghai, China

**Keywords:** Cancer genomics, Non-small-cell lung cancer

## Abstract

Non-small cell lung cancer (NSCLC) remains a leading cause of cancer mortality, and it remains challenging to predict immunotherapy responses. This study integrates RNA sequencing data from five NSCLC immunotherapy cohorts to identify three molecular subtypes, with a copper-dependent proliferation subtype showing poor prognosis and an immunosuppressive tumor microenvironment. We developed a prognostic model that stratifies patients into high- and low-risk groups by a machine learning pipeline combining 101 algorithmic models. The low-risk group exhibited higher immune infiltration and better progression-free survival, characterized by activation of immune-related pathways, such as IL-2/STAT5 and IFN-γ signaling. CEACAM5^+^ epithelial cells were identified as a high-risk subgroup linked to poorer survival and immunotherapy response via mapping the score of the model and clinical information into single-cell sequencing data. Finally, analysis of clinical specimens with different immunotherapy responses confirmed, by western blot and immunohistochemistry, that expression of CEACAM5^+^ epithelial cells related markers was significantly higher in epithelial cells of the non-MPR group compared with the MPR group. Our findings highlight the importance of genes related to cuproptosis and copper hemostasis as biomarkers for immunotherapy prediction and prognosis stratification.

## Introduction

Lung cancer is the most common malignant tumor worldwide, with the leading mortality rate among all cancer types^1^. Non-small cell lung cancer (NSCLC) is the predominant pathological subtype, accounting for approximately 85% of lung cancer cases. Traditional treatments for NSCLC include surgical resection, chemotherapy, and radiation therapy. However, due to the nonspecific nature of chemotherapy and radiation, these treatments often cause side effects such as bone marrow suppression and gastrointestinal reactions. With the continuous exploration of precision medicine, targeted therapy directed at mutation hotspots has become an important treatment modality for NSCLC^2^. When sensitive mutations are absent, immunotherapy has become another crucial approach for NSCLC treatment, showing improvements in efficacy and tolerability compared to conventional chemotherapy and radiation therapy^3,4^. In recent years, several forms of cell death that could precisely induce death of tumor cells have gained widespread attention, offering potential new targets for precision therapy in NSCLC.

Cuproptosis is a novel form of cell death dependent on copper ions and mitochondrial respiration. Tsvetkov et al. have identified ten key genes closely related to cuproptosis by a genome-wide CRISPR knockout screen, which play crucial roles in the process of cuproptosis. For example, the FDX1 gene exhibits significant cytotoxicity and functional enhancement by reducing copper ions from divalent to monovalent copper^5^. Similarly, CDKN2A, GLS, and MTF1 genes were shown to be decisive for cell sensitivity to cuproptosis^6^. Further studies revealed that genes such as SLC31A1, ATP7A, and ATP7B influenced the cell death process by regulating intracellular copper ion concentration^6^. Among them, SLC31A1 is responsible for the intracellular transport of copper ions, while the ATP7A and ATP7B genes are involved in the extracellular excretion of copper ions, which together constitute an important mechanism of cellular copper ion transport and directly affect the intra- and extracellular homeostasis of copper^7–9^. A recent study demonstrated that Zinc transporter 1 (ZnT1, encoded by SLC30A1), as a novel copper ion transporter, mediates the entry of copper ions into cells and induces cuproptosis^10^. Therefore, these genes are also considered as cuproptosis key genes (CKGs), including CDKN2A, FDX1, DLD, DLAT, LIAS, GLS, LIPT1, MTF1, PDHA1, PDHB, ATP7A, ATP7B, SLC30A1, and SLC31A1.

With the deeper exploration of CKGs within the field of oncology, more and more studies have begun to focus on the expression of these genes in different tumor types and their potential prognostic value. Several studies have confirmed the role of CKGs, as well as long non-coding RNAs (lncRNAs) associated with cuproptosis, including in NSCLC^11–14^. In addition, the association between tumor immune microenvironment (TIME) and CKGs has attracted extensive attention. Early studies have revealed a close link between TIME and a series of regulatory cell death processes, including apoptosis, ferroptosis, pyroptosis, necrosis, and autophagy^15–17^. Induction of these inflammatory forms of cell death in the tumor environment may trigger the release of damage-associated molecular patterns (DAMPs) and specific cytokines, which in turn modulate the function of innate and adaptive immune cells involved in anti-tumor immune responses. Furthermore, molecular subtypes based on ferroptosis^18^, pyroptosis^19,20^, and necrosis^21^ have been developed and help predict the prognosis and the efficacy of immunotherapy.

Nevertheless, the potential links between CKGs and the immune microenvironment of non-small cell lung cancer (NSCLC), as well as their use as an effective model for predicting immunotherapy prognosis, are still unclear. Therefore, this study aimed to explore the association between CKGs and NSCLC prognosis and immune microenvironment characteristics, and to establish a prognostic gene signature by using a machine learning model, so as to further explore the association between cuproptosis and NSCLC in depth, and to provide some references for the precise diagnosis and treatment of NSCLC.

## Results

**Fig. 1:**
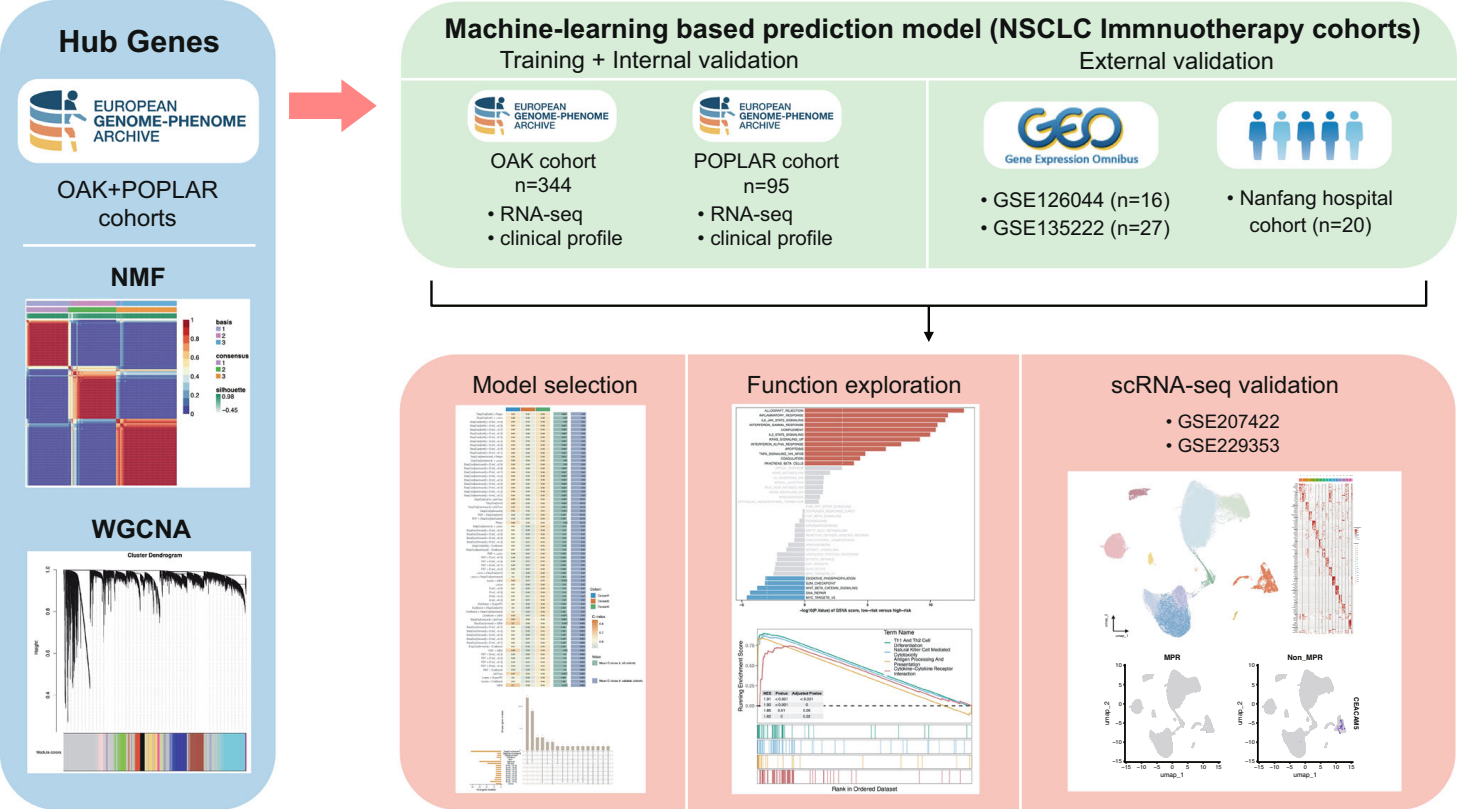
Overview of the study design and methodology. First, identification of hub genes was conducted using the OAK and POPLAR cohorts from the European Genome-phenome Archive (EGA). Gene expression data were analyzed using Non-negative Matrix Factorization (NMF) and Weighted Gene Co-expression Network Analysis (WGCNA). Subsequently, development of a machine-learning-based prediction model for NSCLC immunotherapy cohorts was undertaken. The model was trained and internally validated using RNA-seq and clinical profiles from the OAK (*n* = 344) and POPLAR (*n* = 95) cohorts, with external validation conducted using datasets from GSE126044 (*n* = 16) and GSE135222 (*n* = 27), as well as a Nanfang hospital cohort (*n* = 20). Finally, model selection and functional exploration were conducted, with survival analysis, pathway enrichment, and gene expression profiling visualized. Additionally, single-cell RNA sequencing validation was performed using GSE207422 and GSE229353 to further explore immune cell populations in NSCLC.

The workflow of this study is shown in (Fig. 1). To investigate the relationship between cuproptosis and immunotherapy in NSCLC, we included 439 of 891 cases in OAK and POPLAR cohorts, who underwent immunotherapy rather than chemotherapy only.

### NSCLC cuproptosis subclusters and characteristics

**Fig. 2:**
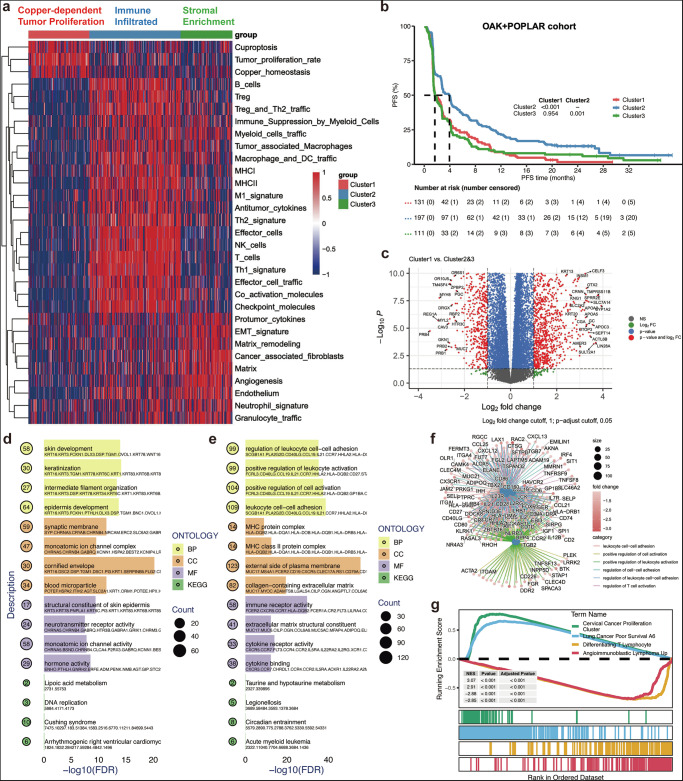
NSCLC subclusters and characteristics related to cuproptosis and immune pathways. **a** Heatmap of gene expression across 31 pathways: The heatmap displays the expression patterns of three main clusters related to copper-dependent tumor proliferation, immune infiltration, and stromal enrichment across three distinct clusters (Cluster 1, Cluster 2, Cluster 3) in the OAK + POPLAR cohort. The color scale indicates the degree of expression, with blue representing low expression and red representing high expression. Categories such as Treg, B cells, immune response markers, and matrix remodeling are highlighted, providing insight into the differential gene signatures in these groups. **b** Kaplan–Meier survival analysis: The survival curve compares the progression-free survival (PFS) between the three clusters in the OAK and POPLAR cohort. Cluster 1 exhibits a significantly worse PFS compared to Cluster 2 (*p*-value < 0.001). **c** Volcano plot of differential gene expression: This plot shows the differential expression analysis between Cluster 1 and Clusters 2 and 3. Red points indicate significantly upregulated genes, green points indicate significantly downregulated genes, and gray points represent genes with no significant change. **d** Enrichment analysis: The bar plot shows the top enriched biological processes (BP) identified through Gene Ontology (GO) analysis for upregulated genes in cluster1. The *x*-axis represents the significance (−log10(FDR)) of the enriched terms, while the y-axis lists the top GO terms. Terms related to skin development, keratinization, and cell communication are highlighted. **e** Enrichment analysis of downregulated genes in Cluster 1. **f** Network of differentially expressed genes: a network analysis reveals the complex interactions among differentially expressed genes, highlighting key regulatory pathways. The network nodes represent genes, with edges indicating interactions. Enriched biological processes (BP), molecular functions (MF), and KEGG pathways are shown, underscoring the connectivity between immune response, cell adhesion, and tumor progression pathways. **g** Gene set enrichment analysis (GSEA): The GSEA plot illustrates the enriched KEGG pathways in the different clusters. Notably, “Cervical Cancer Proliferation” and “Antigen-activated lymphocytes” are among the top enriched terms, with a significant enrichment score. The term’s ranking in the ordered dataset is shown below the plot.

We performed non-negative matrix factorization (NMF) clustering on the OAK and POPLAR cohorts to identify distinct groups of patients with NSCLC based on the genesets mentioned in the Method section (Fig. S1a, b). The analysis yielded three clusters (*k* = 3), labeled Cluster 1, Cluster 2, and Cluster 3, which exhibited significant differences in 29 immune-related pathways, as well as cuproptosis and copper hemostasis scores (Fig. 2a). Cluster 2 exhibited characteristics of an immune-infiltrated subtype, characterized by significantly high infiltration of immune cells, including T cells, macrophages, and NK cells. This cluster also displayed immune response pathways and cytokine signaling, suggesting a strong immune response profile. In contrast, Cluster 3 represented an immune desert landscape, characterized by an enrichment in stromal components and low immune cell infiltration. This cluster was defined by high expression of genes related to stromal remodeling and immune suppression, consistent with a stroma-enriched immune desert phenotype. Cluster 1, on the other hand, exhibited a prominent tumor proliferation signature, characterized by high levels of genes associated with tumor proliferation. Interestingly, this cluster exhibited significant enrichment in genes associated with cuproptosis and copper homeostasis simultaneously, indicating it as a distinct subtype of copper-dependent tumor proliferation. This subtype may represent a unique group with a potential for targeted therapy based on its copper metabolism dependency. Survival analysis revealed that Cluster 1 had the worst prognosis for PFS, while Cluster 2 showed the best survival outcome, as indicated by the Kaplan-Meier curves (Fig. 2b). The differences in survival between Cluster 1 vs. Cluster 2 (*p* < 0.001) and Cluster 3 vs. Cluster 2 (*p* = 0.001) statistically significant.

Next, we identified differentially expressed genes (DEGs) between Cluster 1 and the other two clusters (Cluster 2 and Cluster 3) (Fig. 2c). A total of 2277 DEGs were identified. To further explore the biological significance of the DEGs, we conducted Gene Ontology (GO) and KEGG pathway enrichment analyses. As shown in Fig. 2d, e, GO terms related to skin development, epidermis development, and epithelial cell differentiation were enriched in Cluster 1. In contrast, immune response-related terms, including leukocyte cell-cell adhesion and positive regulation of leukocyte activation, were downregulated compared to Cluster 2 and Cluster 3. KEGG pathway analysis revealed downregulated genes in Cluster 1 were enriched in pathways related to TIME signaling, including the cytokine-cytokine receptor interaction pathway and T cell regulation (Fig. 2f). Gene Set Enrichment Analysis (GSEA) of MSigDB C2 genesets further supported the distinct biological processes observed in the clusters. In Cluster 1, pathways related cell proliferation and poor survival were enriched (Fig. 2e). These findings suggest that Cluster 1 may represent a less immune-responsive subgroup of NSCLC, associated with poor prognosis and copper-dependent tumor progression.

### Co-expression analysis for screening core genesets

**Fig. 3:**
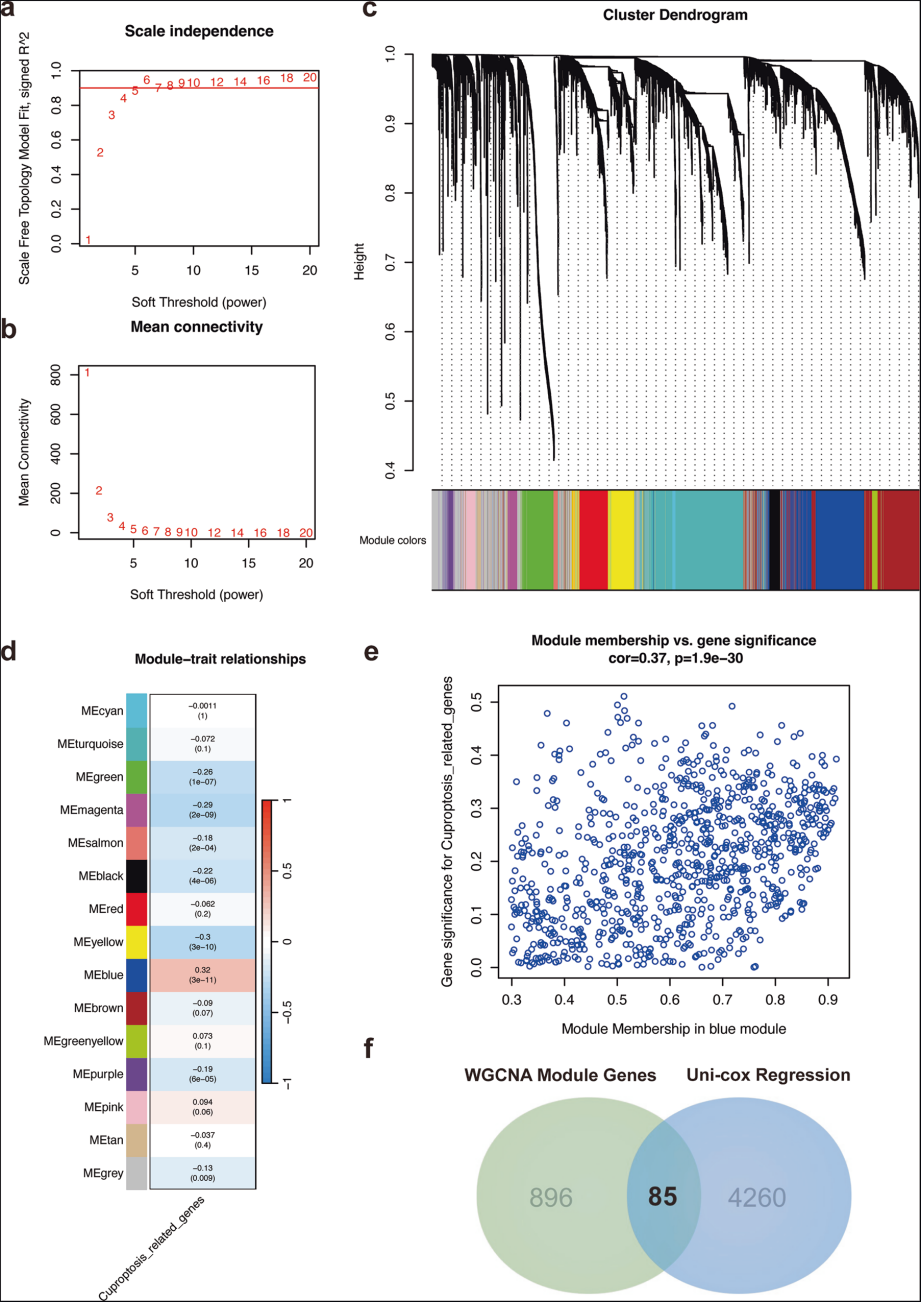
Co-expression analysis for screening core genesets. **a** Scale independence vs. soft threshold (power): The plot shows the relationship between the scale-free topology model fit and the soft threshold (power) used in weighted gene co-expression network analysis (WGCNA). A soft threshold of 6 was chosen based on the highest value, ensuring a scale-free network topology. **b** Mean connectivity vs. soft threshold (power): This plot illustrates the mean connectivity of genes in the network as a function of the soft threshold. A soft threshold of 6 was selected to balance the scale-free topology and mean connectivity. **c** Cluster dendrogram of genes: The hierarchical clustering dendrogram shows the co-expression modules identified by WGCNA. Genes are grouped into modules based on their expression patterns. The module colors are displayed at the bottom, with each color representing a distinct gene module. **d** Module-trait relationships: The heatmap shows the correlation between the identified WGCNA modules (rows) and the trait of interest (Cuptosis-related genes, column). Positive and negative correlations are shown in blue and red, respectively, with statistical significance (p-value) indicated on the right. The blue modules (e.g., MEcyan, MEturquoise) show significant positive correlations, while other modules exhibit weaker or no significant relationships. **e** Module membership vs. gene significance for Cuptosis-related genes: The scatter plot demonstrates the correlation between module membership (i.e., the degree of a gene’s association with a given module) and the gene significance for Cuptosis-related genes. A positive correlation (cor = 0.37, *p* = 1.9e-30) indicates that genes in the blue module are highly associated with the Cuptosis-related trait. **f** Venn diagram of WGCNA module genes and uni-cox regression results: The Venn diagram shows the overlap between the genes identified by WGCNA (896 genes) and those selected through uni-cox regression (4260 genes). The intersection (85 genes) represents genes that are common to both analyses, highlighting potential key genes related to Cuptosis.

To figure out the core gene modules associated with Cluster 1 and cuproptosis, we performed Weighted Gene Co-expression Network Analysis (WGCNA) using DEGs identified from Cluster 1 versus Clusters 2 and 3. We selected an optimal soft-thresholding power to ensure scale-free topology (Fig. 3a) and evaluated the mean connectivity of the network (Fig. 3b). Based on hierarchical clustering, we identified multiple gene co-expression modules represented by different colors (Fig. 3c). To determine the biological relevance of these modules, we correlated them with key functional traits, particularly cuproptosis-related gene signatures. The module-trait heatmap (Fig. 3d) revealed that the blue module showed the strongest positive correlation with cuproptosis-related genes, while other modules exhibited varying degrees of association. A scatter plot of module membership versus gene significance for cuproptosis-related genes (Fig. 3e) further confirmed the enrichment of cuproptosis-associated genes in the blue module (correlation = 0.37, *p* < 0.0001), suggesting a potential role in the copper-dependent tumor proliferation phenotype observed in Cluster 1. To identify clinically relevant hub genes, we intersected WGCNA module genes with significant prognostic genes derived from univariate Cox regression analysis, which yielded 85 hub genes serving as potential biomarkers or therapeutic targets for NSCLC patients with a copper-dependent tumor proliferation subtype (Fig. 3f).

### Constructing machine learning prediction models based on hubgenes

**Fig. 4:**
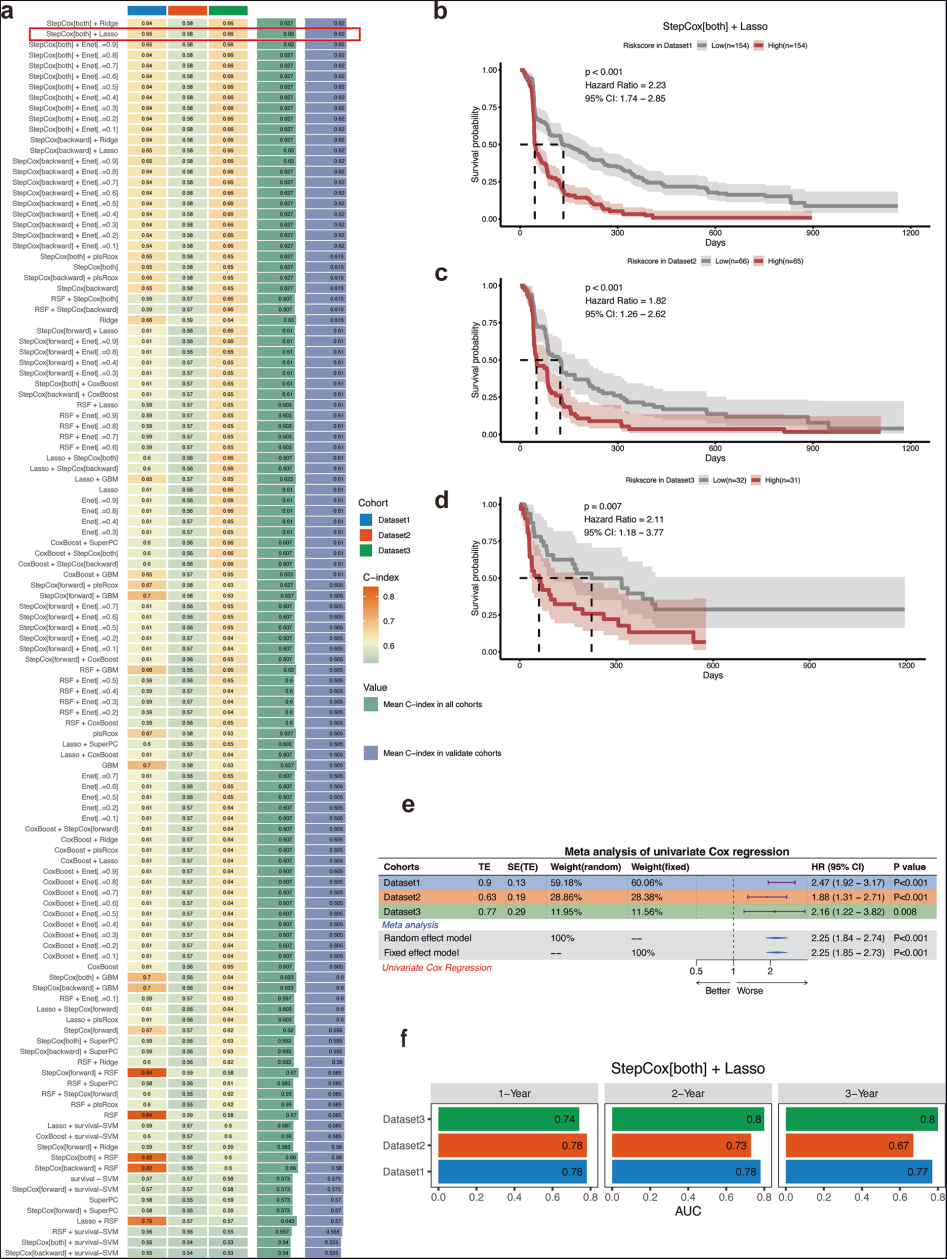
Constructing machine learning prediction models based on hubgenes. **a** Performance of 101 machine learning models: This table presents the evaluation of 101 machine learning models, including various regression and classification algorithms, based on their concordance index (C-index) across multiple datasets. The model StepCox[both] + Lasso (highlighted in the red box) shows the best performance with the highest C-index values in both training and validation cohorts. The table also includes results from other models, such as random survival forests (RSF), support vector machines (SVM), and others, demonstrating their predictive abilities. **b** Kaplan–Meier survival analysis for StepCox[both] + Lasso (Dataset1): The Kaplan–Meier survival curve illustrates the survival probabilities of patients stratified by the predicted risk from the StepCox[both] + Lasso model in Dataset1. High-risk (red) patients show significantly lower survival compared to low-risk (gray) patients (*p* < 0.001, hazard ratio = 2.23, 95% CI: 1.74–2.65). **c** Kaplan–Meier survival analysis for StepCox[both] + Lasso (Dataset2): The survival analysis for StepCox[both] + Lasso in Dataset2 shows similar trends, with high-risk patients (red) having worse survival outcomes compared to low-risk patients (gray) (*p* < 0.001, hazard ratio = 1.82, 95% CI: 1.26–2.62). **d** Kaplan–Meier survival analysis for StepCox[both] + Lasso (Dataset3): In Dataset3, the survival analysis shows a statistically significant difference between high-risk and low-risk groups, with a *p*-value of 0.007 and Hazard Ratio = 2.11 (95% CI: 1.18–3.77), indicating the robustness of the StepCox[both] + Lasso model across datasets. **e** Meta-analysis of univariate Cox regression: This meta-analysis table summarizes the hazard ratios (HR) and *p*-values from univariate Cox regression across three datasets. The model StepCox[both] + Lasso shows consistent results across all cohorts, with a pooled hazard ratio of 2.25 (95% CI: 1.68–2.73), demonstrating strong prognostic ability across different populations. **f** 1-, 2-, and 3-year survival prediction AUC: The bar plots show the Area Under the Curve (AUC) for survival prediction at 1, 2, and 3 years using the StepCox[both] + Lasso model across three datasets. The model achieves high AUC values, with 1-year survival AUC = 0.74, 2-year AUC = 0.73, and 3-year AUC = 0.68 in Dataset1, indicating reliable performance in long-term survival prediction.

With the aim to construct Cluster1 feature markers with better prognostic value and immunophenotyping, we utilized a combination of 101 machine learning algorithms to analyze. All the five cohorts were integrated with combat algorithm to remove the batch effect (Fig. S2a, b). The EGA dataset was partitioned into a training set and an internal validation set in a ratio of 1:1. In the training set, we used a ten-fold cross-validation framework, fitted 101 predictive models, and computed C-indexes for all training and validation datasets, as shown in Fig. 4a. Among these 101 models, the top 10 predictive models ranked according to the average C index were finally constructed using the StepCox combined with Ridge regression, Lasso and Enet algorithm. Among them, StepCox[both] plus Lasso regression showed better prediction ability in both the training dataset, and the external validation dataset. In conclusion, after a thorough screening, we identified StepCox[both] + Lasso as a predictive model with high accuracy and prognostic relevance.

Meanwhile, the prediction efficacy of the model was tested on the training set and validation set. The StepCox[both] + Lasso model performed well on both the training set and external validation set (Fig. 4b–d), presenting a significantly high hazard ratio based on the risk score (Fig. 4e). Moreover, the selected model also functioned well in 1-year, 2-year, and 3-year PFS prediction (Fig. 4f).

### Immune and functional enrichment analysis based on risk score

**Fig. 5:**
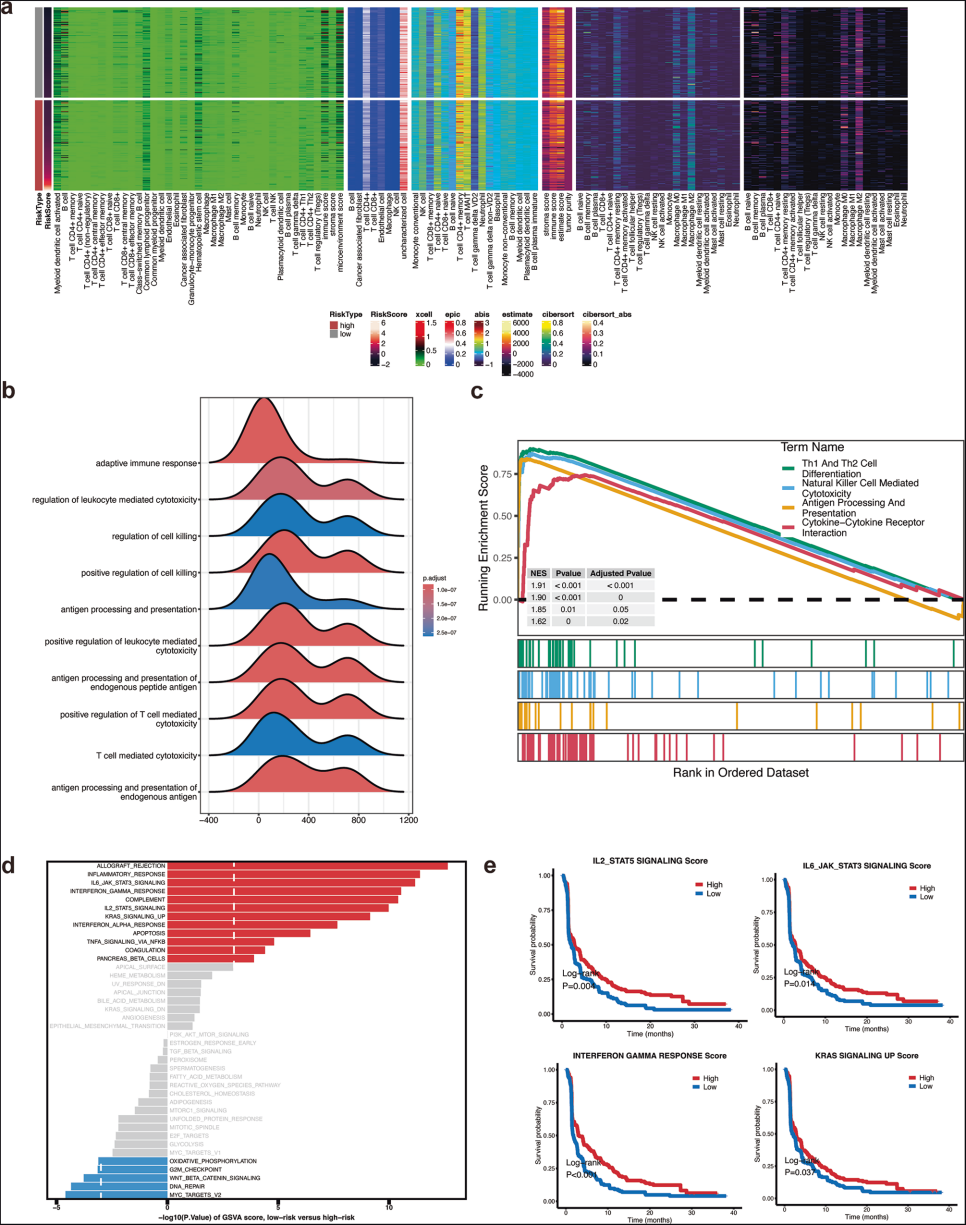
Immune and functional enrichment analysis based on risk score. **a** Immune infiltration analysis: The heatmap shows the immune cell infiltration patterns in the high-risk and low-risk groups. Each row represents a different immune cell type, and each column corresponds to a sample. The color intensity reflects the level of immune cell infiltration, with higher infiltration in the low-risk group observed for several immune cell types, such as CD4^+^ memory T cells. **b** GO enrichment analysis of upregulated genes in the low-risk group: The violin plots show the distribution of Gene Ontology (GO) terms associated with upregulated genes in the low-risk group. Key terms, such as “adaptive immune response,” “regulation of leukocyte-mediated cytotoxicity,” and “positive regulation of T cell-mediated cytotoxicity,” are enriched in the low-risk group, suggesting a stronger immune response. **c** KEGG GSEA analysis for upregulated genes in the low-risk group: The Gene Set Enrichment Analysis (GSEA) plot shows the running enrichment score (ES) for the top KEGG pathways in the low-risk group. The pathways, including “T cell differentiation” and “cytokine-cytokine receptor interaction,” are significantly enriched, reflecting immune-related processes. **d** MSigDB C7 pathway analysis for upregulated genes in the low-risk group: The bar plot shows the enriched pathways from the MSigDB C7 collection in the low-risk group, with pathways such as “ALLERGY REACTION,” “INTERFERON GAMMA RESPONSE,” and “IMMUNE SYSTEM PROCESS” significantly enriched in upregulated genes in the low-risk group. **e** Survival analysis based on pathway scores from MSigDB C7: The Kaplan–Meier survival curves demonstrate that higher scores for key pathways (e.g., “IL2/STAT5 SIGNALING” and “INTERFERON GAMMA RESPONSE”) in the low-risk group are associated with better survival outcomes, with significant differences between the high and low-score subgroups (log-rank *p*-values < 0.001).

To further validate the clinical relevance of the machine learning model, we conducted an in-depth analysis of immune infiltration and functional enrichment pathways based on the risk groups (low risk vs. high risk) generated by the StepCox[both] + Lasso model. We first analyzed the immune microenvironment in both high- and low-risk groups via xcell, CIBERSORT, estimate and ssGSEA. The heatmap (Fig. 5a) shows the immune cell infiltration profiles across different risk types. The low-risk group exhibited significantly higher immune score and microenvironment score, indicating a more immune-infiltrated microenvironment. To be specific, there was a potentially higher infiltration of B cells and CD4+ memory T cells in the low-risk group, which are typically associated with a more activated and anti-tumor immune response.

As shown in Fig. 5b, we identified several enriched biological processes in both risk groups. For example, the low-risk group exhibited significant enrichment in immune-related pathways, including T cell-mediated cytotoxicity and positive regulation of leukocyte-mediated cytotoxicity, consistent with an anti-tumor tumor microenvironment. KEGG pathway enrichment analysis (Fig. 5c) also demonstrated that low-risk groups were enriched in antigen processing and presentation, suggesting a more active immune response.

We then explored the enrichment of immune-related pathways from the MsigDB C7 database. The analysis revealed that the low-risk group had significant enrichment in IL-6/JAK-STAT3 signaling, interferon-gamma response, and IL2-STAT5 signaling (Fig. 5d). These findings suggest that the low-risk group may have immune activation mechanisms linked to cytokine interaction. Meanwhile, patients with high IL-2/STAT5 signaling scores showed significantly better survival outcomes compared to those with low scores (Fig. 5e, top left). High expression of IL-6/JAK-STAT3, interferon-gamma, and KRAS signaling also correlated with better prognosis, further confirming the active immune nature of the low-risk group (Fig. 5e).

### Single-cell sequencing integrated analysis

**Fig. 6:**
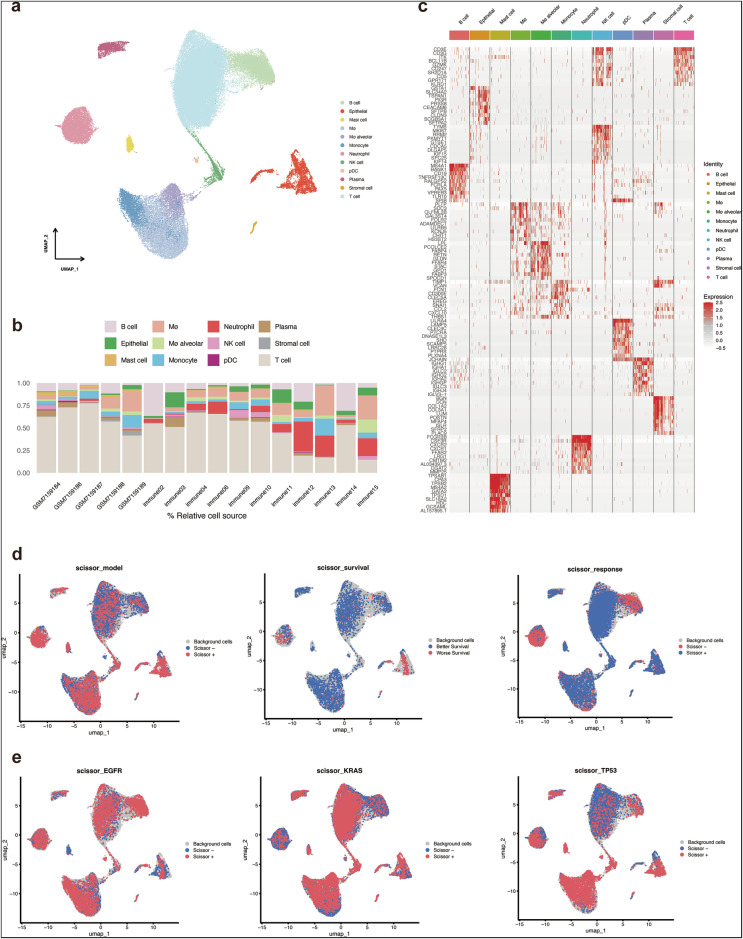
scRNA-seq integrated analysis. **a** Single-cell clustering: The UMAP plot shows the clustering of single-cell RNA-seq data, with different cell types annotated in various colors. Each point represents an individual cell, and the clusters are labeled by the corresponding cell type, including B cells, epithelial cells, macrophages, neutrophils, plasma cells, and T cells, among others. **b** Cell proportion across samples: The stacked bar plot displays the relative proportion of different cell types in each sample. The proportion of each cell type, such as B cells, T cells, and epithelial cells, is represented as a percentage for each sample, providing insights into the cell composition across samples. **c** Cell marker expression: The heatmap illustrates the expression levels of key cell markers for different cell types. Each row represents a marker, and each column represents a different cell type. The heatmap shows high expression of specific markers in their respective cell types, highlighting the cellular identity of each cluster. **d** SCISSOR algorithm – phenotype mapping: The UMAP plot shows the SCISSOR algorithm’s mapping of bulk RNA-seq data onto single-cell data. Cells are colored based on the predicted survival outcome, with blue representing cells associated with better survival and red indicating worse survival outcomes. **e** SCISSOR algorithm—gene mutation mapping: The UMAP plots demonstrate the SCISSOR algorithm’s mapping of bulk RNA-seq data onto single-cell data for various genes. The cells are colored based on gene expression levels for EGFR, KRAS, and TP53 (left to right). Blue indicates lower expression, while red indicates higher expression.

Two NSCLC immunotherapy datasets, GSE207422 and GSE229353 (Fig. 6a–c), were combined and re-analyzed (Fig. S3a–d) to identify specific cell subtypes related to the model we constructed previously using the SCISSOR algorithm to map bulk RNA-seq phenotype data onto scRNA-seq data. It revealed the different distribution of model risk score (scissor_model), survival outcomes (scissor_survival), and therapeutic responses (scissor_response) among different cell subgroups (Fig. 6d). Additionally, the expression patterns of key genetic mutations, including EGFR, KRAS, and TP53, across various cell types were calculated by SCISSOR as well (Fig. 6e). The analysis led to the selection of two specific subgroups, scissor^+^ epithelial and neutrophil, for further investigation. These subgroups were selected based on their significant enrichment in risk score and correlation with the phenotype, including worse survival and less response to immunotherapy.

**Fig. 7:**
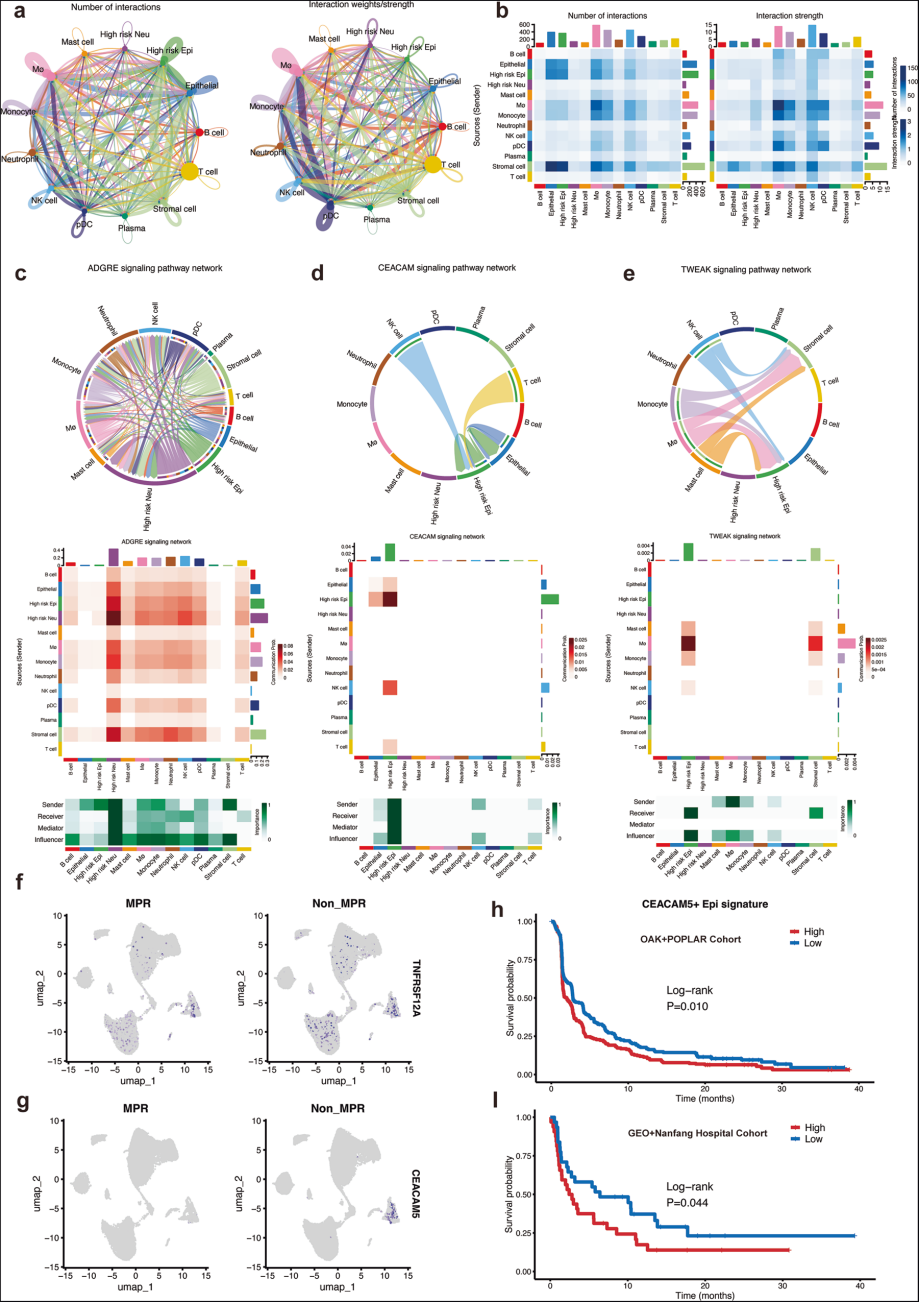
Cell-cell interactions and cell subgroup identified. **a** Cell-cell interaction network: The network plots show the number of interactions (left) and the interaction strength (right) between different cell types, including Mast cells, Neutrophils, Plasma cells, T cells, and others. The interaction edges are colored based on the strength of the interaction, with the size of each node representing the relative abundance of each cell type. **b** Interaction matrix: The heatmaps provide a quantitative representation of the interactions between cell types. The first heatmap (left) shows the number of interactions, while the second heatmap (right) indicates the interaction strength, with darker colors representing stronger interactions. **c** ADGRE signaling pathway network: The circular diagram shows the network of cell-cell interactions involved in the ADGRE signaling pathway, with lines representing interactions between sender, receiver, and mediator cells. The heatmap below visualizes the signaling intensity and interaction frequencies across different cell types for this pathway. **d** CEACAM signaling pathway network: Similar to panel c, the circular diagram displays the interactions within the CEACAM signaling pathway. The heatmap below shows the signaling intensity for each cell type involved, highlighting key interactions. **e** TWEAK signaling pathway network: This panel shows the interactions within the TWEAK signaling pathway, with the circular diagram illustrating the cell types involved in signaling and the heatmap revealing the interaction strength and intensity across cell types. **f** Gene expression in MPR and Non-MPR groups: UMAP plots show the expression of the gene TNFRSF12A in MPR(left) and non-MPR(right) population. The plots reveal significant differential expression between the two groups. **g** Differential expression of CEACAM5: UMAP plots demonstrate the expression of CEACAM5 in MPR and Non-MPR groups. The differential distribution of CEACAM5 indicates its potential role in distinguishing between these two subgroups. **h** Survival analysis based on CEACAM5^+^ epithelial signature: Kaplan–Meier survival curve comparing high and low expression of the CEACAM5^+^ epithelial signature. The log-rank test indicates a significant difference in survival between the high and low groups in OAK + POPLAR cohort (*p* = 0.010) and GEO + Nanfang cohort (*p* = 0.044), with higher expression associated with poorer survival.

In this section, we explored the potential mechanisms underlying the interactions between immune cell subpopulations using CellChat analysis. The network analysis revealed significant communication patterns and interactions between various cell types, including epithelial, macrophages, neutrophils, T cells, and other immune cells in the tumor microenvironment (Fig. 7a, b).

It is observed that the high-risk epithelial and neutrophil subgroups exhibited robust communication patterns, particularly with immune cells such as macrophages and T cells. These interactions were further confirmed through heatmaps that illustrated the frequency and strength of these cellular communications, highlighting the dynamic nature of immune signaling within the tumor. Particularly, we focused on specific signaling pathways that were enriched in these interactions, namely ADGRE, CEACAM, and TWEAK signaling pathways (Fig. 7c–e). Through these pathways, we also identified gene CEACAM5 of CEACAM pathway distinct between the major response (MPR) and Non-MPR subgroups, suggesting the potential roles in modulating immune responses in NSCLC (Fig. 7f, g). Specifically, CEACAM5 expression was found to be enriched in the epithelial subset, supporting its role as a marker for immunotherapy prediction. Furthermore, we investigated the prognostic significance of the CEACAM5^+^ epithelial signature (Fig. S4a–c). Survival analysis revealed that patients with high expression of CEACAM5^+^ epithelial had significantly worse PFS compared to those with low expression in all cohorts (Fig. 7h, i). This result suggests that the CEACAM5^+^ epithelial subgroup may serve as a critical biomarker for stratifying patients based on their immune responses and survival prospects.

### In-vitro experiments validation of CEACAM^+^ epithelial marker genes

**Fig. 8:**
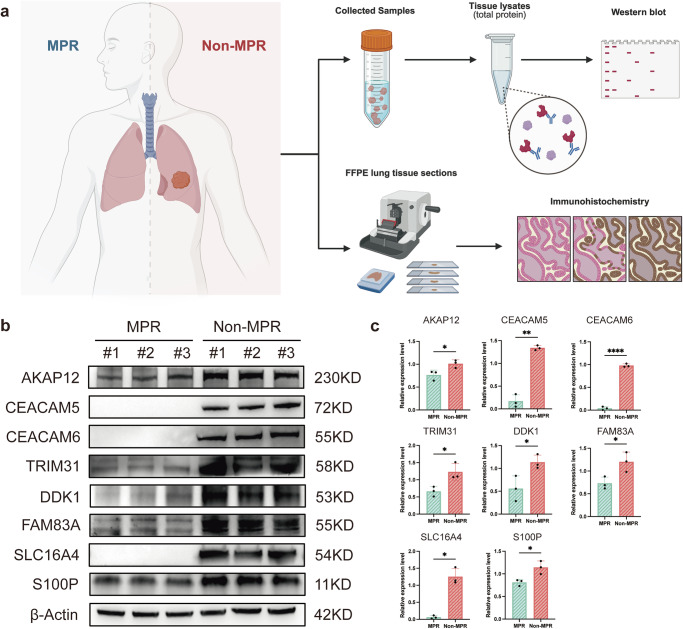
In-vitro experiments and protein expression of CEACAM5^+^ epithelial markers. **a** Clinical samples with different responses to immunotherapy were collected for western blot and Immunohistochemistry. **b** Western blot of 8 key markers and β-Actin as internal control (*n* = 3). **c** Relative expression level based on western blot. **** Means *P* < 0.0001; ** Means *P* < 0.01; * Means *P* < 0.05; ns not significant.

We collected 3 pairs of lung cancer samples with different pathological responses to immunotherapy to validate the differential protein expression of marked genes of CEACAM5^+^ epithelial with western blot and immunohistochemistry (Fig. 8a). The results showed that AKAP12, CEACAM5, CEACAM6, TRIM31, DDK1, FAM83A, SLC16A4, S100P were overexpressed in non-MPR group. The significant difference in expression of these proteins were confirmed through relative expression level (Fig. 8b).

**Fig. 9:**
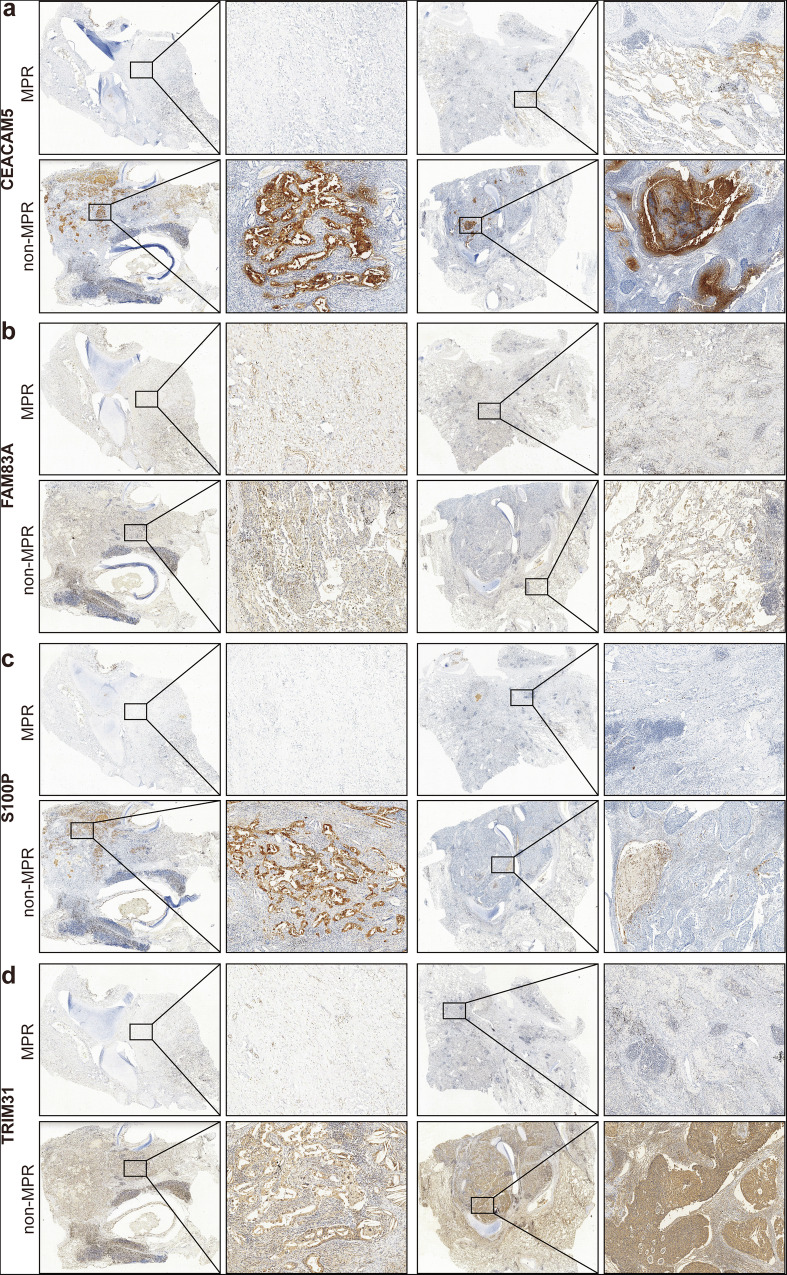
Immunohistochemistry of CEACAM5^+^ epithelial markers. **a** CEACAM5 staining in MPR (up) and non-MPR (down) groups (*n* = 2). **b** FAM83A staining in MPR (up) and non-MPR (down) groups (*n* = 2). **c** S100P staining in MPR (up) and non-MPR (down) groups (*n* = 2). **d** TRIM31 staining in MPR (up) and non-MPR (down) groups (*n* = 2).

To clarify the spatial location of CEACAM5^+^ epithelial markers, immunohistochemistry was conducted in 4 main proteins, including CEACAM5, FAM83A, S100P, and TRIM31 (Fig. 9a–d). The DAB staining demonstrated a higher expression of the four proteins in the non-MPR group. Among them, CEACAM5, S100P, and TRIM31 showed significantly lower expression in the MPR group. Moreover, the staining of CEACAM5 and S100P revealed a potential pattern of co-localization, which indicates the key roles of CEACAM5 and S100P in representing CEACAM5^+^ epithelial cells.

## Discussion

In this study, we used CKGs and copper hemostasis genes to characterize and predict prognostic and immune microenvironmental features of NSCLC patients underwent immunotherapy through multi machine learning models. The results showed that the risk scoring system composed of the characteristic geneset of cuproptosis and copper hemostasis could well stratify NSCLC patients undergoing immunotherapy exhibiting different prognostic and immune infiltration characteristics.

In clinical practice, the use of molecular biomarkers to accurately assess the prognosis of patients with malignant tumors is crucial, a process that involves predicting clinical risk groups and selecting effective treatment strategies, making it a key area of current research^22^. Although significant progress has been made to date in the development and validation of molecular prognostic and/or predictive markers associated with NSCLC, a clear set of specific genes to serve as a reference standard has yet to be identified. In this study, we aimed to explore the molecular prognostic features among different subtypes by selecting cuproptosis-related genes for machine learning analysis and identified a representative set of CKGs. Predictive tools constructed on the basis of these CKGs are not only closely related to the prognosis of NSCLC, but also help us to gain a deeper understanding of the complex biological mechanisms of NSCLC: samples with low-risk scores show a more higher survival, active immunomodulatory pathways, higher immune scores, and richer M1-type macrophage and CD8^+^ T cell^23,24^ infiltration, which play key regulatory and protective roles in the immune microenvironment, whereas samples with high risk scores showed lower survival, active tumor-promoting pathways, high abundance of tumor-promoting immune cells M2-type macrophages, and low immune scores. This may provide some insight into immunotherapy for NSCLC, where immune checkpoint inhibitors (ICIs) may be less effective in patients with a high-risk score.

In our study, we identified CEACAM5^+^ epithelial as the specific cell subtype that was closely related to the prognostic model we conducted. CEACAM5 has been used as a tumor marker for colorectal cancer since 1965 to aid in diagnosis and monitor tumor progression^25,26^. In recent years, with the advancement of research, CEACAM5 has emerged as a promising therapeutic target for the development of new drugs. The Phase I clinical trial (PROCEADE-CRC-01) has brought new hope to patients with advanced CRC: the global first anti-CEACAM5 antibody-drug conjugate (ADC) Precem-TcT has demonstrated significant safety and encouraging efficacy in heavily pretreated metastatic colorectal cancer patients^27^. CEACAM5 is highly expressed in approximately 20% of patients with lung adenocarcinoma. In 2020, Xinwen Zhang et. al. reported elevated CEACAM5 expression in both NSCLC tissues and cell lines. Immunohistochemical (IHC) analysis of tumor samples from 87 patients further revealed that CEACAM5 expression was significantly associated with tumor stage, lymphatic invasion, and histological grade. Moreover, in vitro experiments in this study demonstrated that CEACAM5 can promote NSCLC cell proliferation and migration by inhibiting the p38-SMAD2/3 signaling pathway^28^. Tusamitamab ravtansine (TUSA) is the first antibody-drug conjugate (ADC) targeting CEACAM5. In previous Phase I/II trials (NCT02187848), the drug achieved an objective response rate (ORR) of 20.3% (95% CI: 12.27–31.71%) in a cohort of non-squamous NSCLC patients (*n* = 64) with CEACAM5 expression ≥50%^29^. Anti-CEACAM5 therapy holds promise for broader applications in lung cancer. Based on our findings, CEACAM5 was highly expressed in the non-MPR group, suggesting the potential value of combining anti-CEACAM5 therapy with immunotherapy. However, its specific efficacy requires further confirmation through ethical approval and prospective clinical trials.

Several key challenges remain to be addressed in the use of CKGs to phenocopy the TIME and predict clinical outcomes. First, the role of cell death in TIME may be different for distinct cell types. Given that sensitivity to cuproptosis is dependent on mitochondrial respiration^6^ and upregulation of mitochondrial and tricarboxylic acid cycle metabolism is a prerequisite for the proliferation and function of both cancer cells and T cells^21,30^, both types of cells are susceptible to cuproptosis, but the result may lead to either increased or decreased antitumor activity. In other words, apoptosis in cancer cells and T cells drives response and resistance to ICIs, respectively^31,32^. However, our analysis used pre-treatment bulk-seq data and post-treatment scRNA-seq data, which may not capture the unique contribution of CKGs to TIME in different cells before immunotherapy. It could be further elucidated by analyzing pre-treatment scRNA-seq data with corresponding outcomes of ICIs treatment. Second, cuproptosis may be a double-edged sword like other cell death mechanisms. Depending on the context, cellular pyroptosis and necrotic cell death may promote or inhibit inflammatory responses, ultimately enhancing anti-tumor immunity or promoting tumor growth and metastasis^33^. Third, copper, as a cofactor for enzymes that regulate a wide range of biological processes, also has pros and cons that need to be resolved^34^. In addition to cuproptosis, copper may induce copper proliferative effects that mediate a variety of pro-tumorigenic cellular processes tightly linked to cell proliferation, angiogenesis, and metastasis. Fourth, whether cuproptosis is a predictive or prognostic biomarker has not been confirmed. The data analyzed in this study were obtained from published literature and public databases, which are retrospective studies that require validation through prospective analyses or, in some cases, combined with molecular experiments. Although the different protein expression of the CEACAM5^+^ epithelial marker between MPR and non-MPR groups was confirmed by western blot and immunohistochemistry, the potential mechanism underlying the cuproptosis-related genes and CEACAM5^+^ epithelial requires further investigation.

In conclusion, we identified unique intercellular communication networks and markers based on the cuproptosis and copper hemostasis related genes, such as CEACAM5, which can potentially help stratify NSCLC patients for personalized immunotherapy. The CEACAM5^+^ epithelial signature may serve as a valuable tool for predicting patient survival and tailoring treatment strategies.

## Methods

### Data acquisition and processing

RNA-seq sequence analyses were applied from the European Genome Database for two advanced non-small cell lung cancer immunotherapy clinical studies, the OAK cohort as well as the POPLAR cohort, with samples obtained from pre-treatment surgical/puncture specimens (https://ega-archive.org). One NSCLC cohort with immunotherapy from Nanfang hospital (https://figshare.com/articles/dataset/Nanfang_hospital_NSCLC_immunotherapy_cohort/21564015) and another two immunotherapy cohorts from the Gene Expression Omnibus (GEO) databases (GSE126044, GSE135222) were also included for subsequent analyses, which had complete clinical information and survival data, and were combined and analyzed using the COMBAT algorithm after de-batching. Transcripts per thousand base million (TPM) data from a total of 891 (OAK = 699, POPLAR = 192) patients from the OAK as well as the PAPLAR cohorts were extracted according to previously described methods^35^, and were used as training (dataset1) and internal validation (dataset2) dataset to assess the association of filtered genes with prognosis of immunotherapy. A total of 63 NSCLC patients from the GEO database and Nanfang hospital were included in the external validation dataset (dataset3).

### Composition of genesets

Fourteen genes (CDKN2A, FDX1, DLD, DLAT, LIAS, GLS, LIPT1, MTF1, PDHA1, PDHB, ATP7A, ATP7B, SLC30A1, and SLC31A1) were strongly associated with cuproptosis as previously described and named as CKGs. To improve the predictive efficacy of the model, 32 genes corelated with copper homeostasis and copper metabolism were also included^34^. Meanwhile, to further explore the correlation between cuproptosis and TIME, 29 immune pathway genesets published in previous studies were added to help identify specific cluster as well^36^.

### Unsupervised clustering and co-expression analysis

The NMF package was utilized to identify immunotherapy subtypes and corresponding prognosis^37^. Patients were categorized into three distinct clusters based on the gene expression score of cuproptosis, copper hemostasis, and immune-related pathways via the NMF algorithm. Differential expression was assessed using the DESeq2 package for R (version: 1.42.0). To correct for false-positive results in the expression data, we adopted adjusted P values. The criteria for screening differentially expressed RNA were set as adjusted *P*-value < 0.05 and |fold change| > 1.

Weighted gene co-expression network (WGCNA) was used in this paper to further screen differential genes and involved them into the training of machine learning model, so as to improve the biological significance of the model we generated.

### Functional enrichment analysis

Functional enrichment analyses were performed using Gene Ontology (GO) and Kyoto Encyclopedia of Genes and Genomes (KEGG) methods to compare the differential signaling pathways and biological effects between low- and high-expression cohorts of CKG. GO and KEGG pathways were evaluated using the “clusterProfiler” package in R^38^. Enrichment analysis for GO and KEGG was based on *q*-value and *p*-value thresholds, both set at <0.05.

### Characterization of the immune microenvironment: CIBERSORT, ssGSEA and ESTIMATE

To characterize the immune infiltration in different database, expression data were loaded into CIBERSORT (https://cibersort.stanford.edu/) and repeated 1000 times to determine the relative percentages of 22 immune cell types^39^. Meanwhile, this paper utilizes the ESTIMATE algorithm based on RNA-seq expression levels using the R package “estimate”^40^ The ESTIMATE score, immune score and stroma score of all datasets were calculated using the R package “estimate”, and the immune cell infiltration abundance was obtained by single-sample gene set enrichment analysis (ssGSEA) using the R package “GSVA”, which were combined to validate the immune cell abundance obtained by CIBERSORT.

### Machine learning models

The EGA dataset (OAK, POPLAR cohort) was randomly divided into a training set and an internal validation set according to a 1:1 ratio to ensure a balanced distribution of clinical features between the two groups. The GEO dataset (GSE126044, GSE135222) and Nanfang hospital cohort was set as the external validation set. Integration of including Lasso, Ridge, Stepwise Cox, CoxBoost, Random Survival Forest (RSF), Elastic Networks (Enet), Cox’s Partial Least Squares Regression (plsRcox), Supervised Principal Components (SuperPC), Generalized Augmented Regression Modeling (GBM), and Survival Support Vector Machines (survival-SVM) ten machine learning algorithms. In the training dataset, based on the ten-fold cross-validation framework, we organize 101 combinations of these ten algorithms for variable selection and model construction.

All constructed models were conducted through the package MIME1^41^. For each model, we calculated its C-index in the training set, internal validation set and external validation set. The predictive performance of the models was then ranked based on the average C-index. A combination of algorithms with both robust performance and clinical translational significance was selected, and appropriate model validation methods were chosen based on the best performance.

### Construction and validation of cuproptosis-associated immune gene signature

The filtered geneset obtained from the above model was reintroduced into the final model analysis to create a prognostic risk score in the training dataset. Subsequently, patients were categorized into high-risk and low-risk groups based on the risk score. The difference in PFS between the two groups was assessed using the Kaplan–Meier method. The same formula and statistical analysis were used to verify the prognostic value of geneset in the other datasets.

### Integration of single-cell RNA sequencing datasets

Two single cell RNA sequencing (scRNA-seq) datasets from GEO Database (GSE207422, GSE229353) were integrated with the quality control strategy described in their original article. Cluster specific markers were identified through FindAllMarkers algorithm in Seurat V5.

### SCISSOR analysis

We used SCISSOR^42^ to associate phenotypic data from bulk RNA-seq experiments with single-cell data. Clinical profile and gene expression data of OAK and POPLAR cohorts were obtained from the EGA database. SCISSOR was run on the resolution of each patient individually according to the SCISSOR tutorial using model risk level, immunotherapy response and mutation data (logistic regression), and Progress-free survival (cox-regression) as dependent variables.

### Antibodies

CEACAM5 (CST, 2383 T), CEACAM6 (CST, 85102 T), S100P (PTG, 11803-1-AP), FAM83A (PTG, 20618-1-AP), AKAP12 (PTG, 25199-1-AP), SLC16A4 (PTG, 20889-1-AP), TRIM31 (PTG, 12543-1-AP), DKK1 (PTG, 21112-1-AP).

### Western blot

Tissue samples were homogenized in RIPA buffer (Sigma-Aldrich, St. Louis, MO) containing 1× HALT protease inhibitor (Thermo Fisher Scientific, Waltham, MA) on ice for 30 min. The homogenates were centrifuged at 10,000 rpm for 10 min, and the supernatant was collected. Protein concentrations were quantified using the BCA assay with a BSA standard curve. Equal amounts of protein were mixed with LDS sample buffer and boiled with 10 μM TCEP (Thermo, Cat# 77720).

For insoluble fractions, tissue pellets were resuspended in RIPA buffer with benzonase and processed similarly to the soluble fractions. Proteins were separated by SDS-PAGE on Bis-Tris 4–12% gels and transferred to nitrocellulose membranes. Membranes were blocked for 1–2 h at room temperature in blocking buffer (5% BSA, 0.1% Tween-20 in 1× TBS) and incubated overnight at 4 °C with primary antibodies. After washing with TBST, membranes were incubated with secondary antibodies for 2 h at room temperature before imaging.

### Immunohistochemistry

Paraffin-embedded tissue sections (4 μm thick) were deparaffinized in xylene and rehydrated through a graded ethanol series. Antigen retrieval was performed by incubating slides in 0.01 M citrate buffer (pH 6.0) and heating in a microwave for 15 min. After cooling to room temperature, sections were washed with phosphate-buffered saline (PBS) and blocked with 5% normal goat serum in PBS for 30 min to reduce non-specific binding.

Primary antibodies were applied overnight at 4 °C. The following day, sections were washed with PBS and incubated with appropriate horseradish peroxidase (HRP)-conjugated secondary antibodies for 1 h at room temperature. Immunoreactivity was visualized using the DAB substrate kit (abcam, ab64238), and the staining reaction was monitored under a light microscope. Sections were counterstained with hematoxylin, dehydrated through a graded ethanol series, cleared in xylene, and mounted with a resinous mounting medium. The stained sections were then imaged and analyzed for protein expression.

### Data analysis

All statistical analyses were performed using R (version 4.32). Wilcoxon test was used to analyze variables that were not normally distributed between the two groups. The chi-square test was used to analyze differences in parameters between the high- and low-risk groups. The Kaplan–Meier methodology was used to compare differences in PFS between groups. Univariate Cox regression analysis was performed and presented as risk ratios (HRs) and 95% confidence intervals (CIs) using the “Forestplot” package. In this study, a *P* value of <0.05 was considered statistically significant.

## Supplementary information

Supplementary information

## Data Availability

The datasets used in this paper are available online, as described in the “Methods section”.

## Code availability

No new algorithms were developed for this article. All code generated for analysis is available from the authors upon request.
