# Supplementary material for: Integrative analysis reveals prognostic value of cuproptosis and copper hemostasis related genes in immunotherapy for non-small cell lung cancer
Source: NPJ Precis Oncol. 2025 Nov 20;9:368. doi: 10.1038/s41698-025-01138-7 (PMC12634671; doi:10.1038/s41698-025-01138-7)
Supplement: Supplementary file 1 — Supplementary information [file 41698_2025_1138_MOESM1_ESM.pdf]

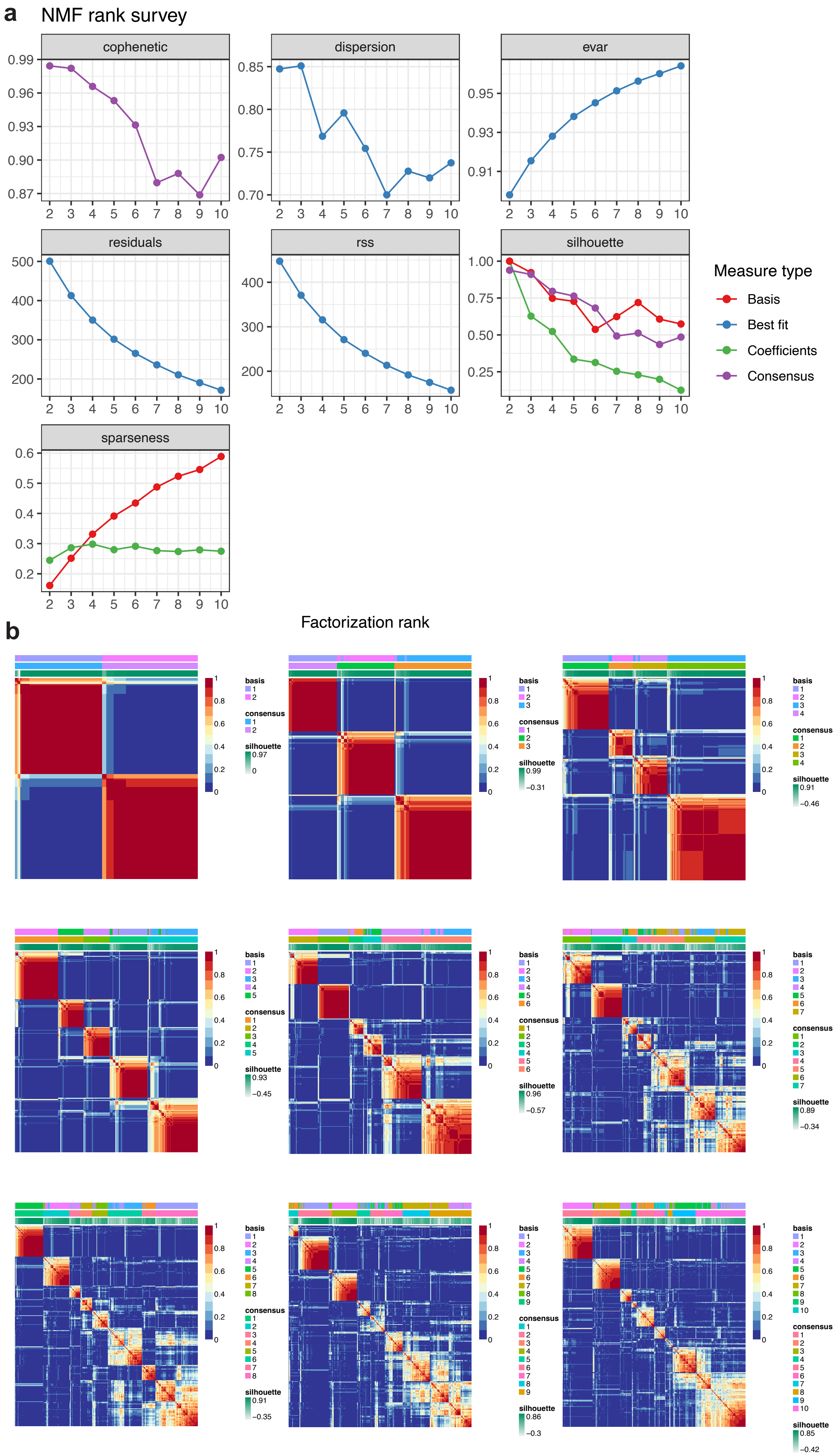

FigS1. Cell-cell interactions and cell subgroup identified

(a) NMF rank survey: results of a non-negative matrix factorization (NMF) rank survey across different ranks (from 2 to 10). Each plot represents different evaluation measure.

(b) Factorization rank: The heatmaps represent the factorization rank results from the NMF procedure. Each square represents the factorization of a different rank, with color intensity reflecting the agreement of clustering based on the selected measures (basis, consensus, silhouette, etc.). Higher values in the heatmaps correspond to better consistency in clustering and more distinct factorization results across ranks.

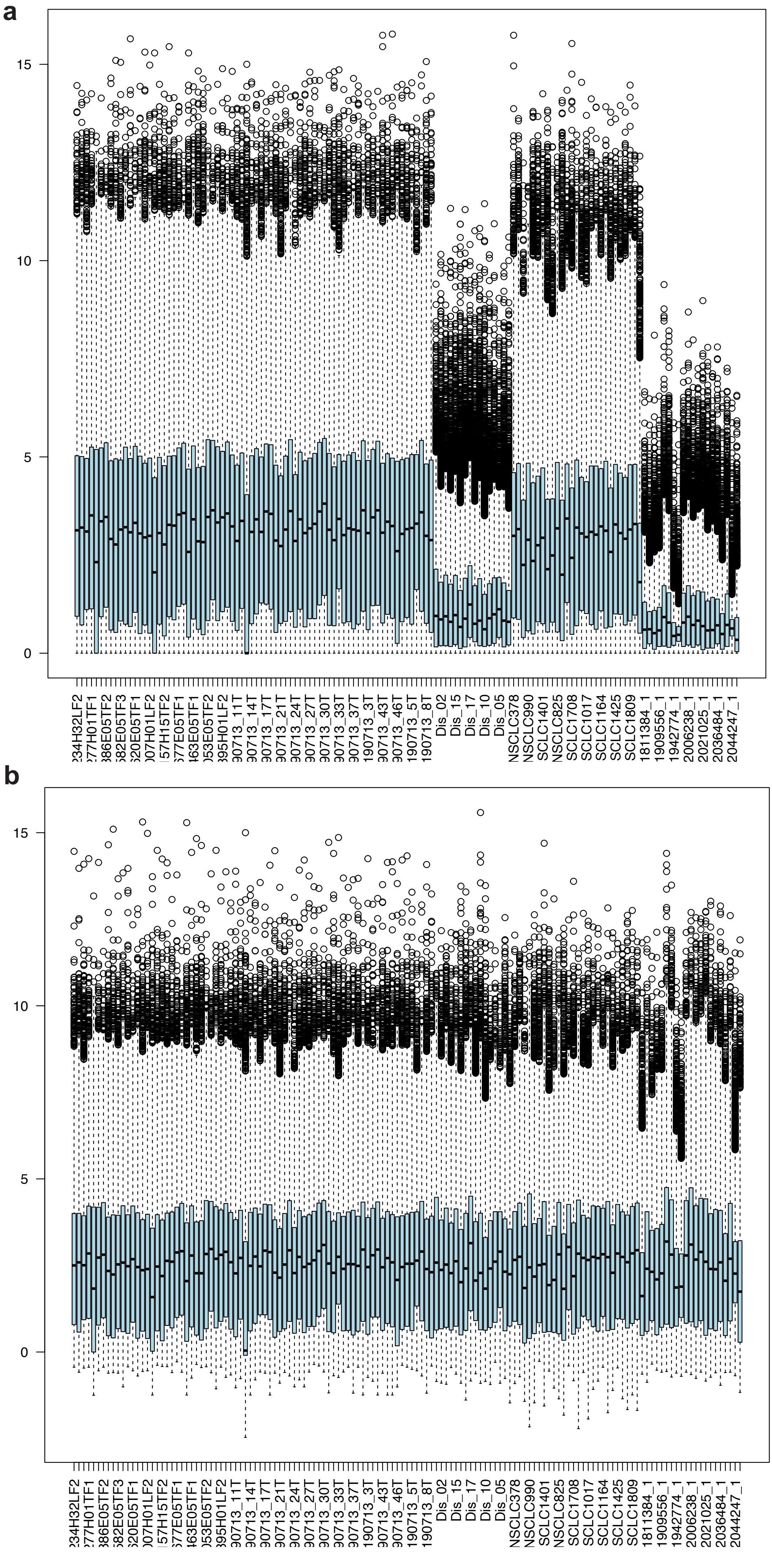

FigS2. Removing batch-effect using comobat algorithm

(a) Before removing batch-effect.

(b) After removing batch-effect.

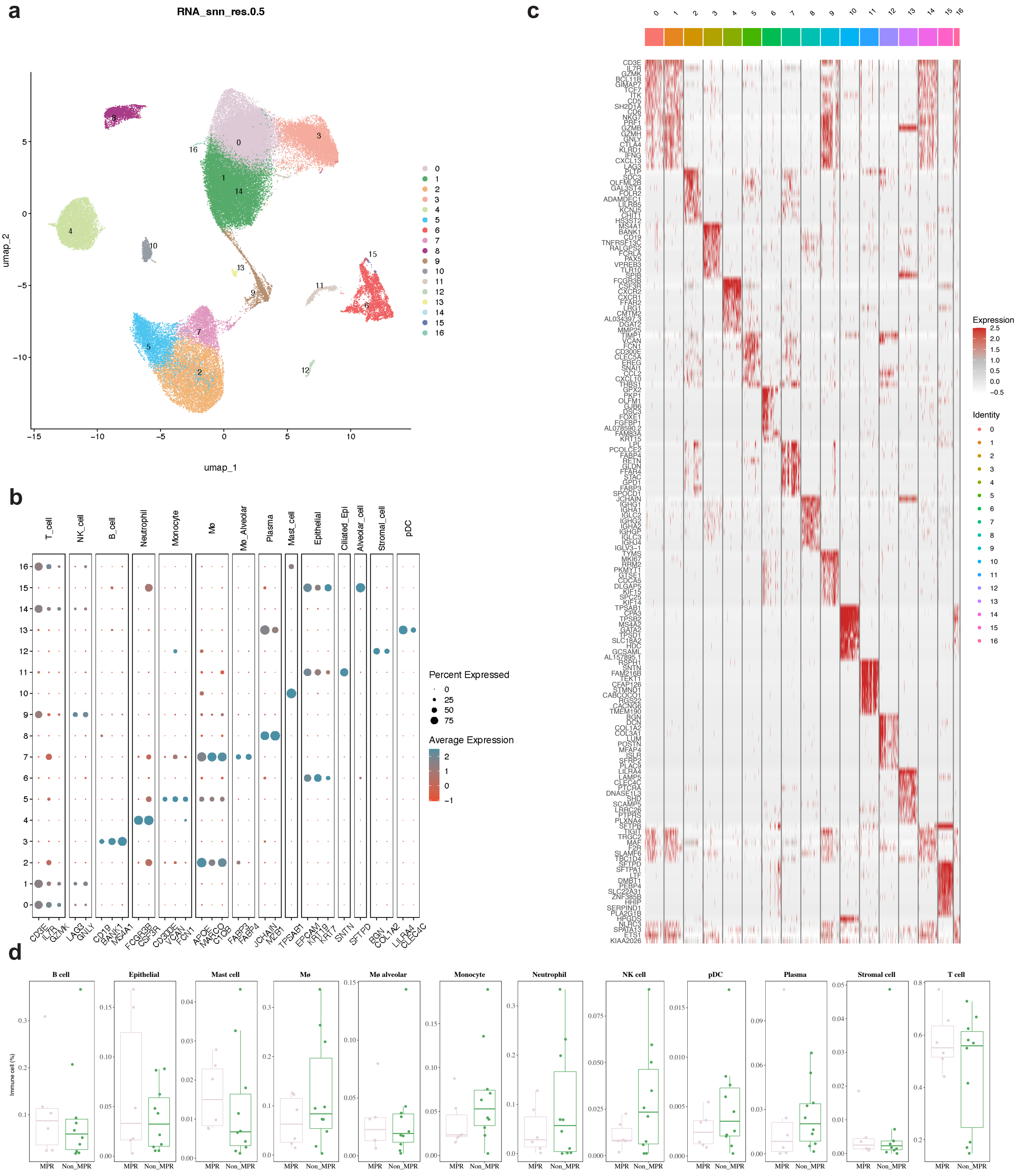

FigS3. Single cell landscape of NSCLC patients with ICIs treatment

(a) Cell clustering under resolution of 0.5.

(b) Cell markers used to distinguish different cell types.

(c) Top 10 genes across 16 cluster.

(d) Different cell portion in MPR and non-MPR goups.

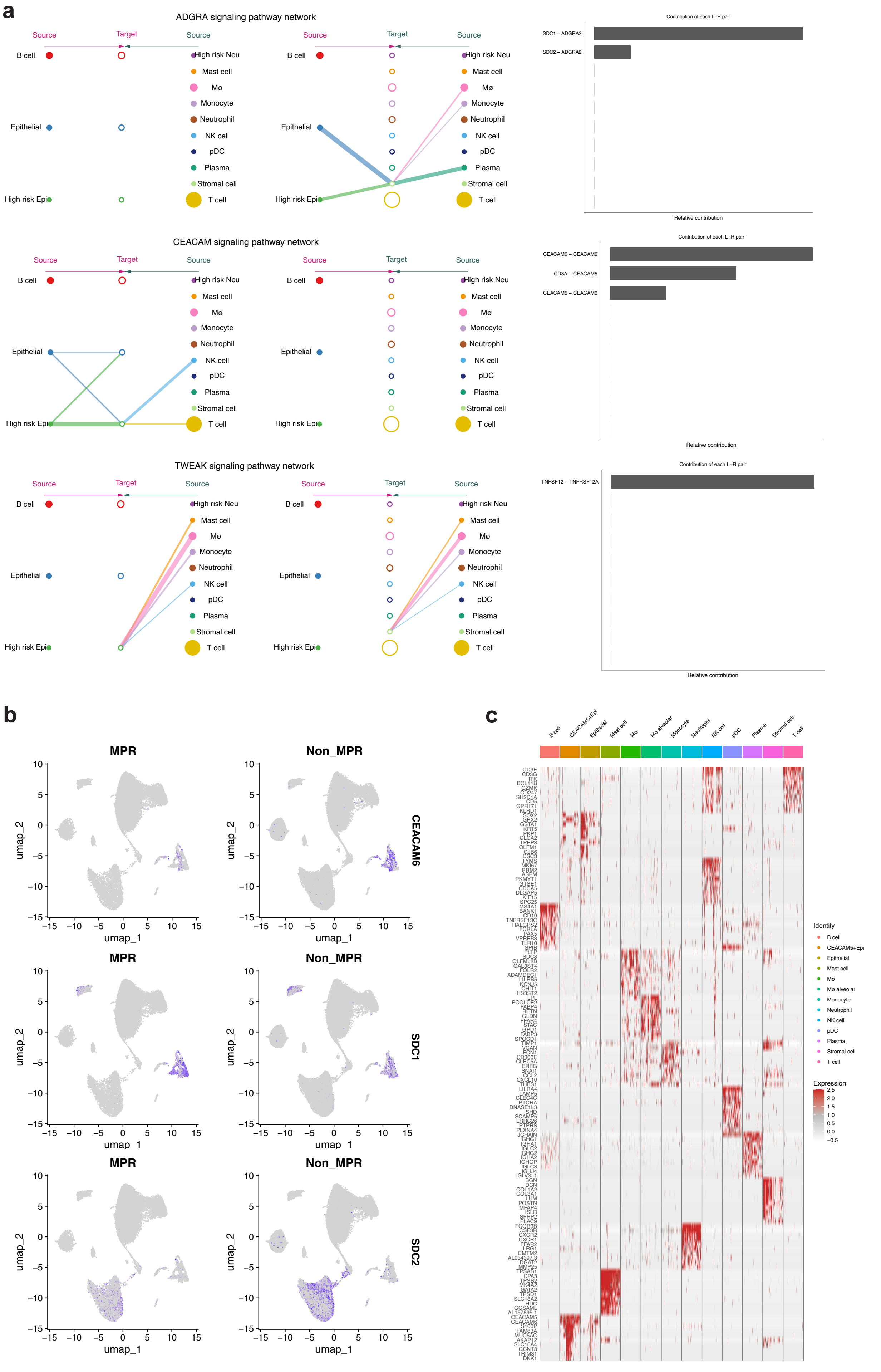

FigS4. Key pathways related to specific cell subgroups and distinct gene signature of CEACAM5+ epithelial.

(a) Key genes of pathways interested.

(b) Distribution of key genes.

(c) CEACAM5+ epithelial gene signature identified by Findallmarkers.
